# A Study of Drug Repurposing to Identify SARS-CoV-2 Main Protease (3CLpro) Inhibitors

**DOI:** 10.3390/ijms23126468

**Published:** 2022-06-09

**Authors:** Seri Jo, Luca Signorile, Suwon Kim, Mi-Sun Kim, Oscar Huertas, Raúl Insa, Núria Reig, Dong Hae Shin

**Affiliations:** 1 College of Pharmacy and Graduates School of Pharmaceutical Sciences, Ewha W. University, Seoul 03760, Korea; seri9388@gmail.com (S.J.); suwon910228@naver.com (S.K.); shfwk31@ewha.ac.kr (M.-S.K.); 2 SOM Innovation Biotech SA., Baldiri Reixac, 4, 08028 Barcelona, Spain; signorile@sombiotech.com (L.S.); huertas@sombiotech.com (O.H.); insa@sombiotech.com (R.I.)

**Keywords:** SARS-CoV-2 3CL protease, drug repurposing, antiviral, fret, inhibitory compounds

## Abstract

The outbreak of coronavirus disease 2019 (COVID-19) caused by severe acute respiratory syndrome coronavirus 2 (SARS-CoV-2) wreaked havoc all over the world. Although vaccines for the disease have recently become available and started to be administered to the population in various countries, there is still a strong and urgent need for treatments to cure COVID-19. One of the safest and fastest strategies is represented by drug repurposing (DRPx). In this study, thirty compounds with known safety profiles were identified from a chemical library of Phase II-and-up compounds through a combination of SOM Biotech’s Artificial Intelligence (AI) technology, SOM^AI^PRO, and in silico docking calculations with third-party software. The selected compounds were then tested in vitro for inhibitory activity against SARS-CoV-2 main protease (3CLpro or Mpro). Of the thirty compounds, three (cynarine, eravacycline, and prexasertib) displayed strong inhibitory activity against SARS-CoV-2 3CLpro. VeroE6 cells infected with SARS-CoV-2 were used to find the cell protection capability of each candidate. Among the three compounds, only eravacycline showed potential antiviral activities with no significant cytotoxicity. A further study is planned for pre-clinical trials.

## 1. Introduction

At the end of December 2019, when a cluster of 27 pneumonia cases with severe acute respiratory syndrome (SARS) of unknown origin emerged in Wuhan [1,2], the general expectation was that the viral disease caused by a new coronavirus (CoV) would have ended like SARS in 2003 and the Middle East respiratory syndrome (MERS) in 2012 [3]. Nobody expected that this disease would dramatically affect the world so quickly and for a long time. By the end of January 2022, more than 300 million people were infected and 5.5 million had deceased, corresponding to a mortality rate of approximately 1.8% (https://covid19.who.int/, WHO Coronavirus (COVID-19) Dashboard, accessed on 1 January 2020). The novel human respiratory coronavirus (SARS-CoV-2) belongs to the genus betacoronavirus [4] and is a positive-sense, single-stranded RNA virus phylogenetically similar to SARS-CoV (~79%) and MERS-CoV (~50%). The most common symptom is fever, followed by cough, dyspnea, myalgia, headache, diarrhea, rhinorrhoea, a sore throat, and pharyngalgia [5]. The pandemic of coronavirus disease 2019 (COVID-19) has stressed the need for treatments, therapeutic alternatives, and vaccines to alleviate and stop the symptoms and to prevent the insurgence of the disease. Currently, among the chemical agents, paxlovid, molnupiravir, and remdesivir have been granted approval for emergency use by the Food and Drug Administration (FDA) to treat hospitalized the outbreak of coronavirus disease 2019 (COVID-19) patients [6,7].

Recently, X-ray crystal structures of the SARS-CoV-2 main protease, also known as the 3-chymotrypsin-like protease (3CLpro) or nsp5 complexed with lead compounds, act-ing as inhibitors have been released [8,9,10,11]. 3CLpro is a cysteine protease whose proteolytic activity is required to process the translation product, vital for viral replication. The sequence identity of 3CLpro between SARS-CoV-2 and SARS-CoV is 96%, with only one different residue in their active sites. Therefore, inhibitory compounds of SARS-CoV are expected to inhibit the SARS-CoV-2 3CLpro, although at different levels. A compound with a vinylsulfone moiety is known to covalently inhibit SARS-CoV 3CLpro [11] and seems to work against SARS-CoV-2 3CLpro as well [12]. These observations have attracted the attention of scientists to quickly find drug candidates to cure COVID-19, using drug repurposing (DRPx) strategies. 

In this study, we assayed thirty compounds selected through an in silico protocol combining a ligand-based virtual screening (VS) method with molecular docking on a Phase II-and-up chemical library. The former uses Artificial Intelligence (AI) and is embodied in SOM Biotech’s technology, SOM^AI^PRO, while the latter was performed through third-party software. Currently, DRPx strategies [13] combined with computational approaches considerably speed up the time to identify promising drug candidates against various diseases. SOM^AI^PRO identifies non-structural analogues of a given compound (the “reference compound”) in a two-step process. SOM^AI^PRO first computes a set of physicochemical characteristics for both the reference compound and for the compounds of the chemical library, and then it uses its AI algorithms to perform pairwise comparisons between the reference compound’s characteristics and those of each compound belonging to the chemical library of choice. SOM Biotech uses a chemical library constituted by compounds in clinical phase II and above. By using a database of compounds that are safe to use in humans, we have been able to identify drugs with antiviral properties that can be quickly moved to clinical trials. In particular, eravacycline was identified at the top positions of the screening, and its inhibitory activity was demonstrated in vitro against recombinant coronavirus 3CL proteases and in VeroE6 cell infection assays. As an approved compound with a good safety profile, eravacycline is ready for pre-clinical trials to test its antiviral potential in COVID-19 patients.

## 2. Results

### 2.1. Identification of Potential SARS-CoV-2 3CLpro Inhibitors by Virtual Screening

The molecules identified from the SOM^AI^PRO screening were ranked according to their potential functional similarity to the reference compound N3, a covalent inhibitor of SARS-CoV-2 3CLpro (PDB 6LU7) [11], and the 300 top-scoring hit compounds were selected for docking calculations. We first proceeded with the validation of the docking workflow by performing covalent redocking of the reference compound N3. The redocked pose was comparable to that of the crystal, with a root mean square deviation (RMSD) of 1.33 Å calculated over the maximum common structure of the ligands and with a docking score (an approximation of the binding free energy ΔG_bind_) of −9.82 kcal/mol. We then applied the same workflow to eight of the top-scoring 300 molecules as ranked by SOM^AI^PRO; in fact, only eight molecules could form a covalent bond with Cys145, through a Michael addition reaction. Next, in order to identify potential non-covalent inhibitors, we set out to perform non-covalent docking with the remaining 292 compounds. We first validated the non-covalent docking workflow by redocking the ligand N3; a pose similar to the crystallized compound N3 was obtained, with an RMSD of 1.17 Å over the maximum common structure of the ligands and with a non-covalent redocking score of −11.31 kcal/mol. Finally, we applied the same workflow to the remaining 292 compounds that could not form a covalent bond with Cys145. Among the molecules with the best docking scores, obtained from both covalent and non-covalent docking, a total of 30 compounds were short-listed after visual pose inspection and according to their in silico scores, the feasibility of development, and druggability for the new indication (COVID-19), in order to be tested experimentally.

### 2.2. Identification of Potential SARS-CoV-2 3CLpro Inhibitors by In Vitro Assay

**Figure 1:**
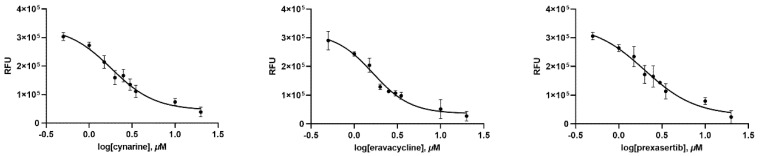
Inhibition of SARS-CoV-2 3CLpro. Each data point represents the effect of each inhibitory compound against SARS-CoV-2 3CLpro compared with the control. The RFU are plotted against the log-concentration of inhibitory compounds. Each dot is expressed as the mean ± standard error of the mean (*n* = 3). RFU = relative fluorescence units.

In order to test whether the top thirty compounds have inhibitory activity against SARS-CoV-2 3CLpro, an inhibitory effect of each compound at 20 μM was tested. Intriguingly, three compounds, cynarine, eravacycline, and prexasertib, showed a prominent inhibitory activity against SARS-CoV-2 3CLpro. The binding affinity data were plotted as log(inhibitor) concentration versus fluorescence (Figure 1). Cynarine, eravacycline, and prexasertib presented a strong inhibitory activity with IC_50_ values of 1.815 μM, 1.645 μM, and 1.996 μM respectively. In order to deduce the inhibitory mechanisms of the three compounds, the k_inact_/K_I_ values were evaluated. The k_inact_/K_I_ is a rate constant that describes the efficiency of covalent bond formation attributable to the potency (K_I_) of the first reversible binding event and the maximum potential (k_inact_) of inactivation. Those of cynarine and eravacycline were available, and the values were 44.55 s^−1^M^−1^ and 94.47 s^−1^M^−1^, respectively. In this study, we were unable to accurately determine the k_inact_ for prexasertib (Supplementary Figure S1).

### 2.3. Activity on SARS-CoV and MERS 3CL Proteases

**Figure 2:**
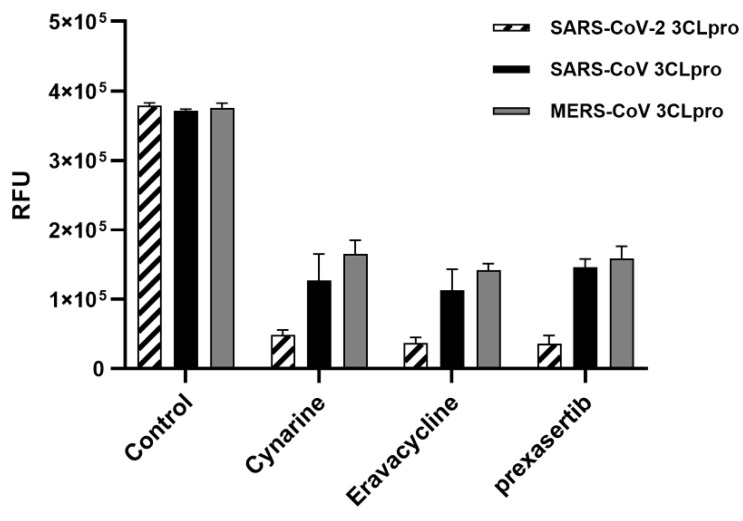
Inhibition of three 3CL proteases. Each column represents the effect of each inhibitory compound against SARS-CoV-2 (left), SARS-CoV (middle), and MERS-CoV (right) 3CLpros compared with the control. All chemicals (20 μM) were confirmed for their inhibitory potential through a comparison of actual absorbance with the control at 490 nm. Data is expressed as the mean ± standard error of the mean (*n* = 3). RFU = relative fluorescence units.

The coronavirus protease 3CLpro is highly conserved in structure and function in all known coronaviruses, as it serves as the main protease for processing of the replicase polyproteins pp1a and pp1ab. There is more than 80% of sequence identity among 3CLpro in coronaviruses from the same genus, and identity reaches 96% between SARS-CoV-2 and SARS-CoV 3CL proteases. This suggests that antiviral inhibitors targeting this protease should have wide-spectrum activity against coronaviruses. We tested the activity of the three covalent inhibitors against SARS-CoV and MERS-CoV 3CL proteases, and as expected, they showed a significant inhibitory activity (Figure 2).

### 2.4. Evaluation of Antiviral Activities of Three Compounds against SARS-CoV-2

**Figure 3:**
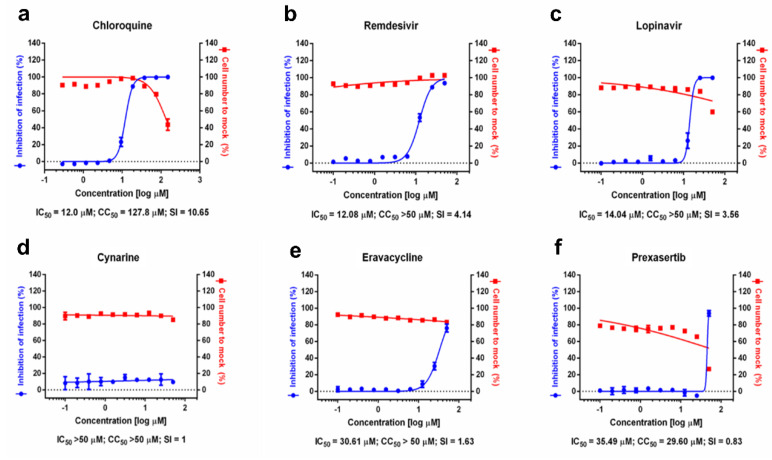
Inhibition of SARS-CoV-2 infection in VeroE6 cells (**a–f**). The blue squares represent inhibition of SARS-CoV-2 infection (%), and the red triangles represent cell viability (%). Mean standard deviation (SD) was calculated from duplicate experiments.

The 3CLprotease is essential for the life cycle of coronaviruses in infected cells, as it mediates the release of the viral proteins that make the machinery for viral transcription, replication, and assembly of new viruses. The antiviral activities of the three positive compounds were tested in SARS-CoV-2 infected VeroE6 cells. The cells were treated with different concentrations of test compounds for one hour prior to the infection with SARS-CoV-2 at a multiplicity of infection of 0.0125. Cell infection was measured 24 h after by viral nucleocapsid protein N staining, and the cytotoxicity of the compounds was also tested in parallel in the absence of virus. As shown in Figure 3, eravacycline inhibited cell infection with an IC_50_ of 30.61 µM without affecting cell viability. Cynarine did not show antiviral or cytotoxic activity, whereas prexasertib was cytotoxic at high concentrations. In comparison, remdesivir, chloroquine, and lopinavir inhibited infection with an IC_50_ of approximately 12, 12, and 14 µM respectively.

### 2.5. Cell Infection Analysis by Immunofluorescent Method

The inhibitory test in VeroE6 cells were performed. The dose–response curves (DRC) for chloroquine, lopinavir, and remdesivir were used as reference drugs as described in the discussion section. Antiviral activities against SARS-CoV-2 together with cytotoxic activity were also included in Figure 3.

## 3. Discussion

**Figure 4:**
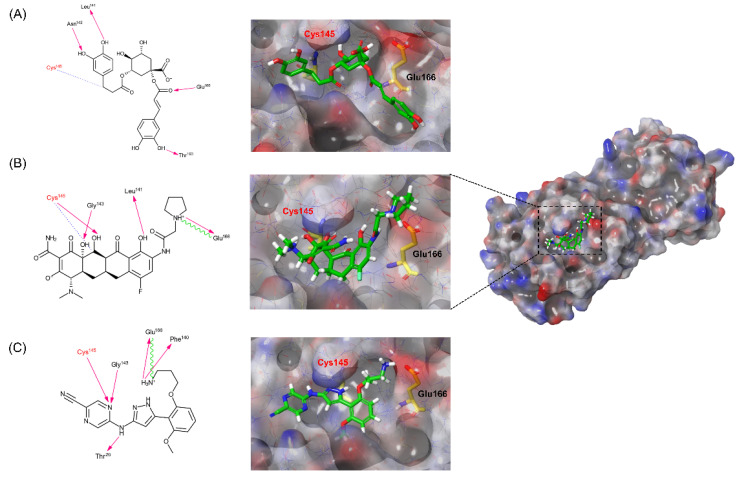
A 2D schematic representation of the interactions between and schematic representations of cynarine (**A**), eravacycline (**B**), and prexasertib (**C**) docked on the catalytic cavity of SARS-CoV-2 3CLpro. The pink arrows represent hydrogen bonds, the blue dot line represents the covalent bond, and the green line represents a salt bridge. The electrostatic surface potential of SARS-CoV-2 3CLpro docked with compounds is depicted (red, negative; blue, positive; white, uncharged).

Drug repositioning (DRPx) has been attracting much attention lately. In fact, the shorter times to enter the market and the lower costs (compared with the development of new drugs) associated with DRPx benefit both pharmaceutical industries and patients. In this study, some of the compounds intended for other diseases were investigated for their potential inhibitory activity on SARS-CoV-2 3CLpro through VS using SOM Biotech’s technology, SOM^AI^PRO, an AI-based VS technology. Approximately 9000 FDA- and Investigational New Drug (IND)-approved drugs were subjected to the VS. Among them, a total of 30 candidates (Supplementary Table S1) were selected. The thirty compounds were applied to an in vitro assay against SARS-CoV-2 3CLpro. Cynarine, eravacycline, and prexasertib represented outstanding inhibitory activity with IC_50_ values of 1.82 *μ*M, 1.65 μM, and 1.99 μM, respectively. Interestingly, eravacycline was also identified in recent virtual screens of FDA-approved drugs for repurposing as COVID-19 treatments [14,15]. The docking modes of cynarine, eravacycline, and prexasertib indicated that between the two catalytic dyad residues His41 and Cys145, Cys145 was involved in binding with all three chemicals (Figure 4). The residue Glu166 in the active site was also predicted as a key residue through hydrogen bonds and a salt bridge interactions. Interestingly, cynarine and eravacycline were expected to form a covalent bond with Cys145 (Figure 4A,B). Therefore, a kinetic study was performed to prove the presence of the irreversible covalent bond. The k_inact_/K_I_ values were evaluated and were 44.55 s^−1^M^−1^ for cynarine and 94.47 s^−1^M^−1^ for eravacycline, respectively. In contrast, the kinetic study of prexasertib showed its competitive, non-covalent inhibitory mode (Figure 4C). The results support the prediction of the binding modes of the three compounds.

The positive compounds were also capable of inhibiting SARS and MERS 3CLproteases as expected, given the highly conserved sequence of the 3CLpro among coronavirus. These results suggest that these compounds may have broad antiviral activity against betacoronavirus (Figure 2).

To test the capacity of these compounds to inhibit viral infection and replication in cells, VeroE6 cells were pre-treated with each candidate prior to infection with a pathogenic strain of SARS-CoV-2. The infected cells were scored by immunofluorescence analysis, and the confocal microscope images of both the viral N protein and cell nuclei were analyzed. The dose–response curve (DRC) for each drug was generated. Chloroquine, lopinavir, and remdesivir were used as reference drugs, with 50% inhibitory concentration (IC_50_) values of 12.0, 14.04, and 12.08 μM, respectively (Figure 3a–c). Among the 3 chemicals that were evaluated in our study, only eravacycline showed potential antiviral activities against SARS-CoV-2, with IC_50_ values of 30.61 μM and no significant cytotoxic activity in the conditions used (Figure 3e). Eravacycline is a synthetic fluorocycline antibiotic of Tetraphase Pharmaceuticals, which possesses a broad spectrum of activity against gram-negative, gram-positive aerobic, and facultative bacteria, including multi-drug-resistant strains [16]. It is a safe drug that has been approved by FDA and EMA for the treatment of complicated intra-abdominal infections, currently commercialized in the US under the brand name of Xerava. It is administered by intravenous infusion twice a day, and it is known to accumulate in the lungs at concentrations that are compatible with its in vitro activity against SARS-CoV-2 3CLpro [17]. This, together with its known antibacterial activity that could help prevent secondary infections in hospitalized COVID-19 patients and the known anti-inflammatory actions of tetracyclines [18], warrants further research on the potential role of eravacycline for the treatment of patients with COVID-19 or infected with SARS-CoV-2 but with milder symptoms. Since the current intravenous formulation may limit eravacycline’s use in non-hospitalized patients, the development of oral formulations would be highly beneficial. In addition, it is known that tetracyclines (and therefore eravacycline) are metal chelating agents and that metal chelation can interfere with their absorption [19], thus likely decreasing eravacycline’s bioavailability toward SARS-CoV-2 3CLpro in vivo. However, as an example, metal chelation was found to affect tetracycline’s antibiotic activity differently in Staphylococcus aureus (where the metal-bound tetracycline was more active) and Escherichia coli (where tetracycline in its free form was more active) [20]. It would be interesting to assess whether metals can modulate eravacycline’s inhibitory activity toward the SARS-CoV-2 3CLpro, as is the case for the different antibiotic activity between Staphylococcus aureus and Escherichia coli mentioned before. It should be pointed out that no metal ions were present in the crystal structure used for the in silico studies presented here. It is worth noting that prexasertib caused cell toxicity in this setting. It was developed by Eli Lilly to cure ovarian cancer patients by targeting CHEK1, a serine/threonine-specific protein kinase [21]. The cell toxicity may be the consequence of the inactivation of CHEK1, affecting the initiation of cell cycle checkpoints, cell cycle arrest, and DNA repair. Cynarine is a natural compound found in artichoke leaves with reported choleretic properties [22]. The inefficiency of cynarine may be related to reactivity of a carboxyl center that can be easily modified by the surrounding environment [23]. In fact, cynarine is quickly metabolized after oral administration [24], and the activity of its metabolites against Mpro should be determined to assess its potential role in the treatment of COVID-19. Moreover, both prexasertib and cynarine might also have metal-chelating properties like those of eravacycline, which again could affect their bioavailability in vivo. Therefore, we propose that eravacycline is a potent candidate to cure patients with COVID-19, while the other two compounds are good therapeutic candidates only if modified properly.

## 4. Materials and Methods

### 4.1. 3CLpro Protein Expression and Purification

The coding sequence of SARS-CoV-2 3C-like (chymotrypsin-like) protease (3CLpro) (NCBI Ref. seq. YP_009725301.1) was synthesized chemically by Bioneer (Daedeok-gu, Daejeon, Korea) and cloned into a bacteriophage T7-based expression vector. The coding sequence of SARS-CoV nsp5-pp1a/pp1ab 3CLpro (NCBI Ref. seq. NP_828863.1) was synthesized chemically by Bioneer (Daedeok-gu, Daejeon, Korea) and cloned into a bacteriophage T7-based expression vector. The coding sequence of MERS-CoV nsp5, 3CLpro (NCBI Ref. seq. NC_019843.3) was synthesized chemically by Bioneer and cloned into a bacteriophage T7-based expression vector. The plasmid DNAs were transformed into *E. coli* BL21 (DE3) for protein expression. *E. coli* BL21 (DE3) cells were grown on Luria–Bertani (LB) agar plates containing 150 μg mL^−1^ ampicillin. Several colonies were picked and grown in capped test tubes with 10 mL LB broth containing 150 μg mL^−1^ ampicillin. Cell stocks composed of 0.85 mL culture and 0.15 mL glycerol were prepared and frozen at 193 K for use in large cultures. The frozen cell stocks were grown in 5 mL LB medium and diluted into 1000 mL fresh LB medium. The cultures were incubated at 310 K by shaking until an OD_600_ of 0.6–0.8 was reached. At this point, the expression of 3CLpro was induced using isopropyl-β-D-1-thiogalactopyranoside (IPTG) at a final concentration of 1 mM. The cultures were further grown at 310 K for 3 h in a shaking incubator. Cells were harvested by centrifugation at 7650× *g* (6500 rev min^−1^) for 10 min in a high-speed refrigerated centrifuge at 277 K. The cultured cell paste was resuspended in 30 mL of a buffer consisting of 50 mM Tris–HCl pH 8.0, 100 mM NaCl, 10 mM imidazole, 1 mM phenylmethylsulfonyl fluoride (PMSF), and 10 μg mL^−1^ DNase I. The cell suspensions were disrupted using an ultrasonic cell disruptor (Digital Sonifier 450, Branson, MO, USA). Cell debris were pelleted by centrifugation at 24,900× *g* (15,000 rev min^−1^) for 30 min in a high-speed refrigerated ultra-centrifuge at 277 K. 

SARS-CoV-2 3CLpro was purified by cation chromatography using a 5 mL Hi-Trap Q column (GE Healthcare, Piscataway, NJ, USA). The column was equilibrated with a buffer consisting of 20 mM Tris pH 7.5, and the pooled fractions were loaded. The column was eluted using a linear NaCl gradient to 1.0 M NaCl, and the protein was eluted at 0.18 M NaCl. 

SARS-CoV 3CLpro was purified by cation chromatography using a 5 mL Hi-Trap Q column (GE Healthcare, Piscataway, NJ, USA). The column was equilibrated with a buffer consisting of 20 mM Tris pH 8.0, and the pooled fractions were loaded. The column was eluted using a linear NaCl gradient to 1.0 M NaCl, and the protein was eluted at 0.28 M NaCl. 

MERS 3CLpro was purified by cation chromatography using a 5 mL Hi-Trap Q column (GE Healthcare, Chicago, IL, USA), followed by a 5 mL Hi-Trap Blue column (GE Healthcare, Chicago, IL, USA).

### 4.2. FRET Protease Assays with SARS-CoV-2, SARS-CoV, and MERS 3CL Proteases

The enzyme activity assays were previously described [25]. The custom-synthesized fluorogenic substrate, DABCYL-KTSAVLQSGFRKME-EDANS (ANYGEN, Gwangju, Korea), was used as a substrate for the proteolytic assay using the SARS-CoV-2, SARS-CoV, and MERS 3CL proteases [26,27]. This substrate contains the nsp4/nsp5 cleavage sequence, GVLQ↓SG [27], and works as a generic peptide substrate for many coronaviruses. The peptide was dissolved in distilled water and incubated with each protease. A SpectraMax i3x Multi-mode microplate reader (Molecular Devices, San Jose, CA, USA) was used to measure spectral-based fluorescence. The proteolytic activity was determined at 310 K by following the increase in fluorescence (λ_excitation_ = 340 nm, λ_emission_ = 490 nm, bandwidths = 9, 15 nm, respectively) of EDANS upon peptide hydrolysis as a function of time. Assays were conducted in black 96-well plates (Nunc) in 300 μL assay buffers containing protease and substrate as follows. For the SARS-CoV-2 3CLpro assay, 2.04 μL of 0.294 mM protease containing 20 mM Tris pH 7.5 was incubated with 7.5 μL of 0.1 mM substrate at 310 K for 2 h 30 min before measuring relative fluorescence units (RFU). Before the assay, the emission spectra of antiviral agents and some of their adjuvants were surveyed after illuminating at 340 nm to avoid the overlapping with the emission spectrum of EDANS. Every compound was suitable to be tested. The final concentration of the protease, peptide, and chemical used in the assay was 2 μM, 2.5 μM, and 20 μM each. First, the SARS-CoV-2 3CLpro and chemical were mixed and pre-incubated at room temperature for 1 h. The reaction was initiated by the addition of the substrate, and each well was incubated at 310 K for 2 h 30 min. After that, we measured the fluorescence of the mixture in the black 96-well plate using the endpoint mode of SpectraMax i3x, where the excitation wavelength was fixed to 340 nm and the emission wavelength was set to 490 nm using 9 and 15 nm bandwidths, respectively. All reactions were carried out in triplicate.

For the SARS-CoV 3CLpro and the MERS-CoV 3CLpro assay, the final concentrations of the protease, peptide, and chemical used at the assay were 1 μM, 2.5 μM, and 20 μM each. The reaction was initiated by the addition of the substrate, and each well was incubated at 310 K for 2 h.

Among the thirty chemicals, three of them were picked to further assay at a concentration range of 0.5 μM~80 μM. The IC_50_ value, which is the value causing 50% inhibition of the catalytic activity of the 3CL proteases, was calculated by non-linear regression analysis using GraphPad Prism 7.03 (GraphPad Software, San Diego, CA, USA).

In addition, to support the docking result, an experiment was conducted to obtain the irreversible inhibition constants of the active compounds to the SARS-CoV-2 3CL protease, k_inact_/K_I_. As described above, a proteolytic reaction with 2 µM Mpro and 2.5 µM FRET substrate in 300 µL of reaction buffer was carried out at 310 K for excitation at 340 nm and emission at 490 nm. Each compound was tested at 1 μM to 40 μM. Reactions were monitored every 60 s for 2 h. The progress curves were fit to Equations (1) and (2) as described previously [28,29,30], and it was calculated using Dynafit version 4.08.190 [31,32].

P = (vi/k_obs_) * (1 − exp(−k_obs_*t)) (1)

k_obs_ = k_inact_ * I/(I + K_I_) (2)

### 4.3. Compound Database

A proprietary database was used for the in silico screening. This database included compounds with clinical experience, approved drugs, and drugs in clinical development, in order to avoid compounds with serious side effects that would be unsuitable for repositioning. Furthermore, compounds with a molecular weight below 80 Da or above 1400 Da as well as compounds containing heavy metals and radioactive isotopes were excluded, yielding a total of 9072 molecules.

### 4.4. Virtual Screening for Discovery of New SARS-CoV-2 3CLpro Inhibitors

Virtual screening (VS) calculations were performed by means of SOM Biotech’s non-public proprietary technology SOM^AI^PRO, a ligand-based virtual screening computational tool based on the molecular fields (MFs) similarity between a reference compound and a set of molecules of interest (e.g., a compound database). We exploited SOM^AI^PRO to identify approved drugs with potentially similar or greater inhibitory activity than compound N3 (N-[(5-methylisoxazol-3-yl)carbonyl]alanyl-L-valyl-N~1~-((1R,2Z)-4-(benzyloxy)-4-oxo-1-{[(3R)-2-oxopyrrolidin-3-yl]methyl}but-2-enyl)-L-leucinamide) for repositioning efforts towards SARS-CoV-2 3CLpro. Compound N3 was used as a reference compound in its bioactive 3D conformation based on the crystal structure with PDB code 6LU7 and used for MFs comparison with the previously described database.

MFs characterize the molecules according to their favorable interaction sites, thus allowing a prediction of how molecules might interact with the target [33,34]. Therefore, SOM^AI^PRO is based on the comparison between the MFs of a compound (reference compound) and the MFs of all the compounds present in the database [34]. In general, the reference compound is a drug or compound found to be active towards an indication through a specific mechanism of action (MoA). SOM^AI^PRO aims to identify known, safe, and readily available drugs with the same MoA as the reference compound by calculating the MFs’ similarity and ranking the compounds accordingly. As a consequence, other known drugs originally used for different indications and with different scaffolds can be promptly reprofiled for the new indication of interest.

### 4.5. Protein and Ligand Preparation for In Silico Studies

To perform the steps described below, we used the Schrödinger software, release 2019-4 [35], installed on an Intel^®^ Xeon(R) CPU E5-2609 v4 @ 1.70GHz × 16, 64-bit Linux machine.

### 4.6. Protein Preparation

The protein crystal structure of SARS-CoV-2 3CLpro with PDB code 6LU7 was used. The protein was prepared for docking studies through Schrödinger’s Protein Wizard tool [36]. Missing side chains and loops were modeled with the in-built functionality using Prime [37]; water molecules beyond 3.0 Å from heteroatoms or with less than 3 bonds with non-waters after minimization were removed; protein residue protonation states were assigned with PROPKA [38,39] at pH 7.0. Finally, protein heavy atoms were minimized to convergence to 0.30 Å RMSD, using the OPLS_2005 force field [40].

### 4.7. Ligand Preparation

The compounds were ranked according to their potential functional similarity to the reference compound N3. The top-scoring 300 ligands, as ranked by SOM^AI^PRO, were the prepared with the LigPrep tool [41] at pH 7.0 ± 1.0 using Epik [42,43], including the options for desalting and for tautomer generation. Computation of stereoisomers was performed by retaining specified chiralities.

### 4.8. Docking Studies 

#### 4.8.1. Covalent Redocking

First, the protein structure was edited by removing the covalent bond between the compound N3 and the thiol group of Cys145 of the Mpro. The unbound compound N3 was then prepared with LigPrep [41] at pH 7.0 ± 0.5 using Epik [43] prior to redocking. Next, we set out to redock the reference compound N3 in the binding site to validate the covalent docking workflow, performed with the CovDock covalent docking tool [44]. We selected Cys145 as the reactive residue, and the original pose of compound N3 was used to define the centroid for the grid box center. Michael addition was chosen as the reaction type for the prepared compound N3. Covalent redocking was performed, with an energy cutoff of 2.5 kcal/mol to retain poses for further refinement, up to a maximum of 200 poses.

#### 4.8.2. Non-Covalent Redocking

A non-covalent redocking procedure for the compound N3 was also performed using Glide [45] to identify non-covalent inhibitors. The grid dimensions were 10 Å × 10 Å × 10 Å for the inner box and 35 Å × 35 Å × 35 Å for the outer box, using the original ligand pose to define the box centroid. The Van der Waals radius scaling was set to 1.00 to reproduce the binding pose. Standard precision (SP) settings were selected, including flexible ligand, nitrogen inversions, and ring conformations sampling, with the addition of Epik state penalties to the docking scores.

#### 4.8.3. Docking of the Top-Scoring 300 Compounds

Of the prepared, top-scoring 300 compounds as per SOM^AI^PRO ranking, we identified a subset of eight compounds that were able to form a covalent bond with Cys145 through a Michael addition reaction. We performed the docking calculations through the same workflow used for covalent redocking. Similarly, to identify non-covalent inhibitors among the remaining 292 compounds, we performed non-covalent docking with the Glide program [45] using the same workflow used for non-covalent redocking.

### 4.9. Cell Infection Analysis by Immunofluorescent Method

A 384-tissue culture plate was inoculated with 1.2 × 10^4^ VeroE6 cells per well. Twenty-four hours later, DMSO was used to prepare serial dilutions of the compounds (10 points, with 50 μM or 150 μM as the highest concentration) and added to cells in two sets of duplicates. About an hour after the compound treatment at the BSL3 facility, one set of Vero cells were infected with SARS-CoV-2 at MOI (multiplicity of infection) of 0.0125 and cultured at 310 K for 24 h. The other set of Vero cells treated with compounds were left un-infected to monitor the cytotoxic activity of the compounds. After fixing the cells with 4% paraformaldehyde (PFA), permeabilization was performed. The cells were processed with the anti-SARS-CoV-2 Nucleocapsid (N) antibody as the primary antibody and then treated with Alexa-Fluor-488-conjugated goat anti-rabbit IgG as the secondary antibody. Cell nuclei were stained with Hoechst 33342. Fluorescence images were captured using a large-capacity image analysis device (Opera, Perkin Elmer, Waltham, MA, USA). The acquired images were analyzed using the Image Mining (IM) software, an in-house analysis program. The total number of the cells per well was obtained by counting the number of nuclei stained with Hoechst 33342. The infected cells were counted with the number of cells expressing nucleocapsid protein. The infection ratio was calculated with the ratio of cells expressing nucleocapsid protein to the total number of cells. The infection rate was normalized by setting the average infection rate of wells including uninfected cells (mock) as 0% and wells including infected cells (0.5% DMSO group) without compounds as 100%. The response curves according to the concentration of compounds together with the IC_50_ and CC_50_ values were obtained using the Y = Bottom + (Top Bottom)/(1 + (IC_50_/X)Hillslope) formula running the XLFit 4 (IDBS) software. All the IC_50_ and CC_50_ values were measured by two replicates, and the reliability of the assays was judged with Z’- factor and the % coefficient of variation (%CV). Cell experiments infected with SARS-CoV-2 were performed at Institut Pasteur Korea with the guidelines of the Korea National Institute of Health (KNIH), using enhanced biosafety level 3 (BSL3) containment procedures in laboratories approved for use by the KCDC.

## Data Availability

Not applicable.

## Supplementary Materials

The following supporting information can be downloaded at: https://www.mdpi.com/article/10.3390/ijms23126468/s1.

## Abbreviations

3CLpro	3-chymotrypsin-like cysteine protease
DMSO	Dimethyl sulfoxide
DRPx	Drug repurposing
FRET	Fluorescence resonance energy transfer
MoA	Mechanism of action
MFs	Molecular fields
RNA	Ribonucleic acid
SD	Standard deviation
SP	Standard precision
VS	Virtual screening
