# Supplementary material for: A Study of Drug Repurposing to Identify SARS-CoV-2 Main Protease (3CLpro) Inhibitors
Source: Int J Mol Sci. 2022 Jun 9;23(12):6468. doi: 10.3390/ijms23126468 (PMC9224295; doi:10.3390/ijms23126468)
Supplement: Supplementary file 1 [file ijms-23-06468-s001.zip › ijms-1714710-supplementary.pdf]

A Study of Drug Repurposing to Identify SARS-CoV-2 3CLpro Inhibitors

Seri Jo[1†], Luca Signorile[2†], Suwon Kim[1], Mi-Sun Kim[1], Oscar Huertas[2], Raúl Insa[2], Núria Reig[2*] and Dong Hae Shin[1*]

[1] College of Pharmacy and Graduates School of Pharmaceutical Sciences, Ewha W. University, Seoul, Republic of Korea 03760

[2] SOM Innovation Biotech SA. Baldiri Reixac, 4, 08028 Barcelona, Spain

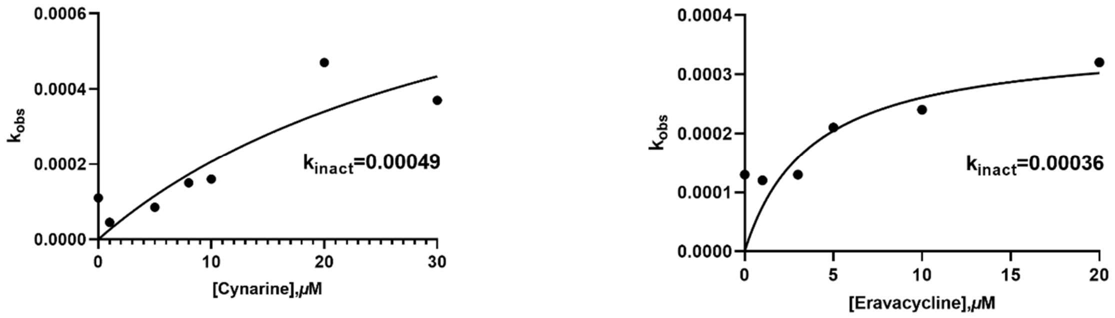

Supplementary Figure S1. In the kinetic studies, 2 μM SARS-CoV-2 3CLpro was added to a solution containing various concentrations of protease inhibitors and 2.5 μM FRET substrate to initiate the reaction. The reaction was then monitored for 2 h. Detailed methods were described in "Materials and methods" section.

Supplementary Table S1. 30 chemical list.

Compound name	CAS	Docking (Covalent)
CYNARIN	30964-13-7	-7,020 (-7,747)
RUPINTRIVIR	223537-30-2	-8,557 (-6,510)
ERAVACYCLINE diHcl	1334714-66-7	-6,458 (-6,090)
TELAPREVIR	402957-28-2	-8,854
COBICISTAT	1004316-88-4	-8,656
BITOLTEROL mesylate	30392-41-7	-8,028
OLCEGEPANT	204697-65-4	-7,832
EPIRUBICIN HCl	56390-09-1	-7,826
VORUCICLIB HCl	1000023-05-1	-7,793
AZIMILIDE diHcl	149888-94-8	-7,642
FLORIFENINE	83863-79-0	-7,597
DIQUAFOSOL TETRASODIUM	211427-08-6	-7,551
UDENAFIL	268203-93-6	-7,415
TAFENOQUINE succinate	106635-81-8	-7,362
DARAPLADIB	356057-34-6	-7,295
LAPATINIB DITOSYLATE	388082-77-7	-7,234
OPROZOMIB	935888-69-0	-7,223
ZARAGOZIC ACID A trisodium	144541-82-2	-7,159
POLDINE METHYLSULFATE	545-80-2	-7,124
PIPEQUALINE	77472-98-1	-7,081
LASMIDITAN HCl	613677-28-4	-6,896
RACTOPAMINE HCl	90274-24-1	-6,878
TOZASERTIB	639089-54-6	-7,208
ETAFENONE HCl	2192-21-4	-6,917
RIMCAZOLE diHCl	75859-03-9	-6,876
PIBENZIMOL	23491-45-4	-5,977
SALMETEROL	89365-50-4	-6,797
PREXASERTIB diHCl	1234015-54-3	-6,558
ABEXINOSTAT	783355-60-2	-6,161
DANUSERTIB	827318-97-8	-5,734
